# Caraway Essential Oil as a Post-Preservative Agent in Low-Salt Cheese Brine

**DOI:** 10.3390/foods14081297

**Published:** 2025-04-08

**Authors:** Neli Ermenlieva, Sylvia Stamova, Kostadin Gramatikov, Sylvia P. Nikolova, Gabriela Tsankova, Emilia Georgieva

**Affiliations:** 1 Department of Microbiology and Virusology, Faculty of Medicine, Medical University, 9000 Varna, Bulgaria; gabriela.tsankova@mu-varna.bg; 2 Department of Pharmaceutical Chemistry, Faculty of Pharmacy, Medical University, 9000 Varna, Bulgaria; 3 Research Institute, Medical University, 9000 Varna, Bulgaria; k.gramatikov@mu-varna.bg; 4 Department of Social Medicine and Health Care Organization, Faculty of Public Health, Medical University, 9000 Varna, Bulgaria; silviya.p.nikolova@gmail.com; 5 Training Sector “Medical Laboratory Technician”, Medical College—Varna, Medical University, 9000 Varna, Bulgaria; emiliya.georgieva@mu-varna.bg

**Keywords:** white brined cheese, *Carum carvi* L., natural preservatives, microbial safety

## Abstract

White brined cheeses, particularly Bulgarian white brined cheese, are emblematic of the Balkans and northeastern Mediterranean culinary traditions, characterized by aging in brine to ensure microbial safety and distinctive sensory qualities. *Carum carvi* L. (caraway), a plant renowned for its aromatic profile, is gaining recognition for its antimicrobial properties. This study evaluated the efficacy of caraway essential oil (rich in carvone, 69.8%, and limonene, 28.19%) as a natural preservative in brine and white brined cheese, focusing on its antimicrobial activity against *Escherichia coli* and *Staphylococcus aureus*. The results demonstrated that at a concentration of 0.12% (*v*/*v*), caraway essential oil effectively inhibited microbial growth, completely suppressing *E. coli* even under high contamination loads and significantly reducing *S. aureus* within 24 h. The minimum bactericidal concentration was identified as 0.25% (*v*/*v*) in both cheese and brine. Importantly, organoleptic assessments confirmed that the essential oil did not compromise sensory qualities, with the cheese maintaining a perfect score of 100 points. These findings underscore the potential of caraway essential oil as a natural preservative for cheese production and storage, offering dual benefits of microbial safety and sensory integrity. Its incorporation aligns with growing consumer demand for natural and sustainable food preservation methods, supporting its application in the dairy industry.

## 1. Introduction

Cheese is an ancient dairy product with a long history of production, traditionally consumed either fresh or fermented. Its unique taste, texture, and high nutritional value have made it one of the most popular foods worldwide [1]. Cheese comes in a wide variety of types, classified based on texture, aging process, and production methods, including fresh, soft, semi-hard, and hard cheeses. A notable category is brined cheeses, which are ripened in a salt solution and widely consumed in various regions. In recent years, international cheese exports have continued to grow for the fifth consecutive year, with a 4.1% increase in 2020, reaching 2.8 million tons, supported by sustained demand from emerging markets [2].

White brined cheeses are widely produced in the northeastern Mediterranean region and the Balkans [3]. Historically, they were made as artisanal cheeses, whereas today they are produced on an industrial scale under strict production controls [4]. These cheeses are rindless, with a slightly sour taste resulting from lactic acid bacteria activity during ripening and a salty taste that develops during brine storage. Therefore, salt and acidity are critical parameters for preserving the physicochemical properties of this type of cheese [5,6]. The low acidity of the cheese creates an unfavorable environment for pathogenic microorganisms, while its relatively high salt content has a similar inhibitory effect on intestinal pathogens. Foodborne pathogens such as *Staphylococcus aureus*, *Escherichia coli* 0157: H7, *Campylobacter jejuni*, *Bacillus cereus*, *Listeria monocytogenes*, *Cryptosporidium hominis*, *Salmonella* spp., and *Clostridium botulinum* pose significant concerns for food safety [7,8].

White brined cheeses undergo ripening in brine with a salt concentration (6–10% NaCl), which acts as a selective factor for the microbiota in the cheese [4]. Additionally, salt has significant roles in cheese production, such as modulating the physicochemical and biochemical properties of cheese during ripening and conditioning the sensory characteristics of the final products. However, during the post-preservation period, the high salt content in the brine has mainly preservative properties, allowing the product to be stored in it for up to several months [9,10]. At the same time, excessive consumption of sodium chloride has become a global issue. According to the World Health Organization (WHO), most people consume levels of sodium chloride that are two times higher than the recommended amount [11]. It is estimated that 75% of sodium intake comes from processed foods [12,13]. Therefore, various strategies are being implemented to reduce the salt concentration in several categories of food products, including cheese. Reducing the salt concentration in brine while maintaining its preservative properties is a technological approach that could also offer significant health benefits for consumers’ diets.

The inclusion of preservatives in cheese is approved by the European Commission [EU1129/2011, 2011], but their excessive use can lead to serious health consequences [14]. In recent years, a clear trend has emerged in consumer demand for food products without preservatives or with minimal natural preservatives. This trend has forced the food industry to seek and begin using plant-based preservatives in their production instead of the standard artificial ones.

For the almost 500 decades, naturally occurring plant products always showed a narrative role to society for their effectiveness against several diseases [15]. In this regard, the incorporation of certain natural additives into cheese brine or directly into the cheese during manufacturing, including maturation, is widely discussed [16,17,18,19]. These natural additives, in the form of dried herbs (spices), extracts, or essential oils, are capable of enhancing the sensory qualities of cheese while also exhibiting preservative properties. According to Ritota et al., natural substances that inhibit the growth of pathogenic microbes in cheese include fresh or dried plants such as black and green pepper, ginger, and thyme; essential oils from clove, bark, bay, black cumin, oregano, cinnamon, rosemary, sage, mint, and thyme; and extracts with antimicrobial activity, including water extracts from pine needles and horseradish tree, and ethanol extracts from cress, cinnamon, lemongrass, sage, garlic, oregano, and rosemary [18]. Essential oils and extracts are effective at limiting or inhibiting the growth of harmful bacteria due to their high content of secondary metabolites, such as phenolic compounds, terpenes, isoflavonoids, ketones, aliphatic alcohols, acids, and aldehydes [20,21].

Our attention was drawn to a less commonly discussed plant with known preservative properties. Belonging to the Apiaceae family, *Carum carvi* L. is a popular plant species used in the food industry as a flavoring agent. The preservative effect of caraway has been known since Ancient Egypt; however, modern scientific literature contains only a limited number of studies investigating its antimicrobial properties as a food additive [22,23,24,25,26] or as a preservative in cheese [15,27,28]. Caraway essential oil (EO) is rich in carvone and limonene, which are its two primary components. The quality of caraway essential oil is generally expressed as the percentage of carvone or, more specifically, the ratio of carvone to limonene [29]. Recent research has documented the antibacterial effects of caraway essential oil extracted from its fruits, along with its insecticidal and fungicidal properties, suggesting its potential for broader applications in the future [24,30,31,32]. Other studies have also reported antioxidant and anti-inflammatory activities associated with caraway EO [33]. These findings pave the way for further exploration of caraway’s role in protecting food and feed from spoilage and contamination. Gniewosz et al. propose the use of pullulan films incorporating caraway EO to enhance the microbiological stability of minimally processed foods [34].

As mentioned above, during the post-preservation period, the brine plays a key role in the microbial safety of the cheese. It provides a sustainable antimicrobial barrier for the product itself. The inclusion of caraway essential oil in the composition of the brine has the potential to offer health benefits in two ways—reducing the salt content in the cheese while simultaneously maintaining its preservative effectiveness.

This study aims to evaluate the preservative potential of low-salt brine enriched with caraway EO in inhibiting *S. aureus* and *E. coli* during white brined cheese storage. Additionally, it investigates the impact of caraway EO on the cheese’s organoleptic properties and explores the antimicrobial mechanisms of its main components against *E. coli* through bioinformatics-based network analysis.

## 2. Materials and Methods

### 2.1. Materials

Caraway EO used in this experiment was 100% pure, composed of certified organic constituents, and commercially supplied from do TERRA (Europe). For the purpose of our study, we utilized farm-produced white brined cheese obtained directly from a dairy farmer in the town of Rakovski, southern Bulgaria. The cheese and brine were stored in vacuum packaging. *Escherichia coli* ATCC 25922 and *Staphylococcus aureus* ATCC 29213 strains (MicroSwab, provided by Ridacom, Sofia, Bulgaria) were activated for the purposes of this study through initial plating on Blood agar (HiMedia, provided by Ridacom, Sofia, Bulgaria).

### 2.2. Gas Chromatography–Mass Spectrometry Analysis

In order to identify the component composition of cumin essential oil, a gas chromatographic analysis was carried out using an apparatus consisting of a 7890A gas chromatograph coupled with a flame ionization detector and a 5975C mass spectral detector (Agilent Technologies, Santa Clara, CA, USA). The analysis utilized a Stabilwax column (Restek, Bellefonte, PA, USA) with dimensions of 30 m in length, 0.25 mm in diameter, and a film-coating thickness of 0.25 µm. The temperature program began at 65 °C and increased to 170 °C at a rate of 1.5 °C per minute, with total analysis duration of 70 min. The injector and detector temperature were set at 250 °C, and the FID temperature was maintained at 250 °C. The carrier gases used were hydrogen and helium, both with a flow rate of 0.8 mL/min. The mass spectral detector operated within a scanning range of *m*/*z* = 40–450, and the sample injection volume was 1.0 µL in a 100:1 flow split model. Component identification was achieved by comparing retention times and relative Kovach indices (RI) with standard substances, as well as through mass spectral data from the NIST’08 (National Institute of Standards and Technology, USA) and Adams Library.

### 2.3. Investigation of the Antimicrobial Activity of Caraway Essential Oil in Brine and Cheese

To initiate this study, cheese brine was prepared using sterile distilled water with a salt concentration lower than that typically found in white brined cheese, specifically 20 g/L (2% salt content) (NaCl, ≥99.0%, Merck Darmstadt, Germany). This brine was used in the preparation of all the samples described below.

To investigate the preservative properties of caraway EO, we prepared four sets, each containing seven samples, with 20 mL of brine and seven different concentrations of caraway EO: 0.06% (*v*/*v*), 0.12% (*v*/*v*), 0.25% (*v*/*v*), 0.5% (*v*/*v*), 1% (*v*/*v*), 2.5% (*v*/*v*), and 5% (*v*/*v*). In all test solutions, 5 g of cheese was added, ensuring complete submersion in the solution, along with 0.1 mL of a standardized microbial suspension (0.5 MF). Negative controls, consisting of 20 mL of brine and 5 g of cheese, and positive controls, containing 20 mL brine, 5 g of cheese, and 0.1 mL of a microbial suspension of one of the two test strains, were also included in the experiment. All samples were prepared in triplicate.

Of the four presented sample sets, two were contaminated with *E. coli* ATCC 25922, each stored at a different temperature (4 °C or 37 °C). Similarly, the other two sets were inoculated with a bacterial suspension of *S. aureus* ATCC 29213, cultured under one of the same two temperature conditions. Microbiological analysis was conducted at three time points (3 h, 24 h, and 7 days), including the detection and enumeration of *E. coli* and *S. aureus*.

For the detection and enumeration of β-D-glucuronidase-positive *E. coli*, we followed the guidelines outlined in ISO 16649-2:2014 [35]. The enzyme β-D-glucuronidase is produced by 94–96% of *E. coli* strains [36]. At the control points, the cheese and brine samples were decimal serially diluted in maximum recovery diluent (HiMedia, supplied by Ridacom, Sofia, Bulgaria), ranging from 10^−1^ to 10^−7^. From each prepared dilution, 1 mL was transferred to the bottom of a sterile Petri dish (d = 90 mm). The samples were overlaid with a thin layer of sterile TBX agar (HiMedia, supplied by Ridacom, Sofia, Bulgaria), cooled to 45–50 °C, and gently mixed. The cultivation was carried out at 44 °C for 24 h. Typical for *E. coli* colonies on TBX agar appeared as blue to blue-green color, and, in case of their appearance, they were enumerated (log_10_ CFU/mL or log_10_ CFU/g). A subset of colonies underwent further biochemical identification using an indole test (Bul Bio-NCIPB, Sofia, Bulgaria) and cultivation on Kligler iron agar (KIA) (Bul Bio-NCIPB, Sofia, Bulgaria). With their help, distinguishing characteristics of *E. coli* were detected—indole-positive, glucose-positive, often gas-producing, and lactose-positive.

Detection and enumeration of coagulase-positive staphylococci were performed following ISO 6888-1:2022 instructions [37]. At the control points, from the prepared dilutions ranging from 10^−1^ to 10^−7^, 0.1 mL of each dilution was spread onto the surface of Baird Parker medium (HiMedia, supplied by Ridacom, Sofia, Bulgaria) using a sterile spatula. Samples were incubated at 37 °C for 48 h, with observations made at 24 and 48 h.

The obtained colonies characteristic of *S. aureus* (black with or without an opalescent halo) were enumerated and expressed as log_10_ CFU/mL or log_10_ CFU/g. At least three colonies from each plate were tested for coagulase production. The coagulase test involved culturing colonies in 1 mL of BHI broth (HiMedia, supplied by Ridacom, Sofia, Bulgaria) for 24 h at 37 °C. Subsequently, 0.1 mL of microbial suspension was transferred to 0.5 mL rabbit plasma (Bul Bio-NCIPB, Sofia, Bulgaria) and incubated at 37 °C for 6 h. Samples were examined for coagulation at 2, 4, and 6 h. The presence of a clot indicated a positive reaction, while the absence of change and persistence of liquid plasma indicated a negative result for coagulase-positive staphylococci.

**Figure 1:**
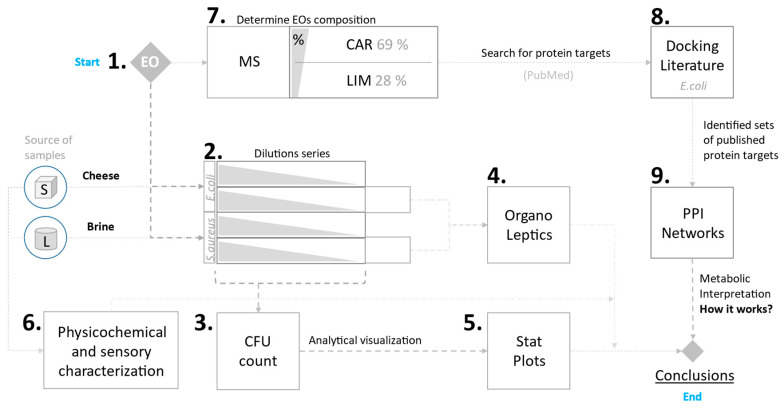
Applied research methods for investigating the preservative potential of brine enriched with caraway essential oil.

The applied research methods described in Section 2 are presented in Figure 1.

### 2.4. Evaluation of the Organoleptic Characteristics of the Samples

The study also aimed to assess the impact of brine containing caraway EO on the organoleptic characteristics of white brined cheese. These characteristics include evaluations of taste, smell, product consistency, appearance of the pieces, cut surface, texture, color, and brine condition, as well as packaging and labeling.

An initial organoleptic assessment of the cheese samples was conducted without the addition of caraway essential oil. Subsequently, cheese samples were added to 20 mL of brine, supplemented with the lowest concentration of caraway essential oil demonstrating antimicrobial activity against both pathogens in our microbiological tests (0.25% *v*/*v*). The samples were stored for 7 days at 4 °C, after which they were assessed using the same 100-point scale and the defined seven parameters. Each parameter was independently evaluated by eight experts, and the average values of their scores were calculated for each characteristic. Each panelist had at least 150 h of prior experience with descriptive analysis of organoleptic properties with various dairy products, including cheese.

### 2.5. Statistical Analysis

To analyze the potential preserving properties of the brine enriched with caraway EO, results from three repeated tests were used. A correlation analysis was performed using Pearson’s correlation coefficient to investigate the relationship between oil concentration and the number of bacterial colonies. The statistical significance of the correlation was assessed using *p*-values, with values below 0.05 considered statistically significant. The analysis was conducted using Jamovi software, version 2.3.

### 2.6. Bioinformatics-Based Network Analysis

An *Escherichia coli* network model was initially seeded with two sets of genes and proteins (nodes) derived from (1) a series of in silico docking studies reported in the literature and retrieved via PubMed [38], and (2) upregulated proteins induced by EOs [39]. This process resulted in a network of twenty-one genes and proteins. The seed network of these twenty-one nodes was then expanded using (1) Ecoli Net, a probabilistic functional gene network tool developed by Yonsei University [40], and (2) additional searches in UniProt [41] and PubMed. This enrichment yielded a final network comprising thirty-five genes and proteins. These thirty-five genes and proteins were then submitted to STRING v12, a database-driven web tool for known and predicted protein–protein interactions (PPIs) [42]. The interactions include both direct (physical) and indirect (functional) associations, derived from computational predictions, knowledge transfer between organisms, and aggregated data from other primary databases. The STRING database currently encompasses 59,309,604 proteins from 12,535 organisms.

Local STRING network clusters were first precomputed, followed by hierarchical clustering of the initial network using an average linkage algorithm. Cluster names were assigned automatically based on consensus protein annotations retrieved from GO, KEGG, BioCyc, Reactome, UniProt, Pfam, SMART, and InterPro. The legend for the inferred clusters is displayed on the right within the frame. To assess PPI interconnectivity within the resulting network, nodes were clustered using the k-means algorithm (set at *c* = 6) to generate distinct functionally related groups (clusters), each with a description and an automatically assigned color, as shown in the figure. Node connectivity within a cluster is represented by solid lines, while inter-cluster edges are depicted as colored dotted lines (default setting). STRING also allows for the assignment of arbitrary colors to protein nodes directly within the generated network.

The final network was exported as an SVG (vector graphic) using the “Export” function, and additional annotations were added in MS PowerPoint.

## 3. Results

### 3.1. Microbial Safety, Organoleptic, and Physicochemical Characteristics of Cheese Samples

Prior to the commencement of this research, the organoleptic and physicochemical characteristics of the cheese used in the study, as well as its microbial safety, were thoroughly examined (Table 1) as part of project No. 22011, funded by the Science Fund of the Medical University of Varna, Bulgaria.

### 3.2. Gas Chromatography–Mass Spectrometry Analysis

Thirteen compounds, representing almost 100% of the total oil volume, were identified in the studied oil (Table 2). The most quantitatively significant components in caraway seed oils were carvone (69.8%) and limonene (28.19%).

### 3.3. Microbiological Analysis at the 3rd Hour After Sample Preparation

The first control point, at which we applied the identification techniques for *E. coli* ATCC 25922 (ISO 16649-2:2014), was at the 3rd hour after sample preparation. Two sets of samples were examined—one stored under refrigerated conditions at 4 °C, suitable for the post-preservation period of food products, and the other stored at 37 °C, providing optimal conditions for microbial growth. Regardless of the two temperature storage regimes, caraway EO demonstrated complete inhibition of microbial growth in all brines and cheese samples containing concentrations ranging from 0.12% (*v*/*v*) to 5% (*v*/*v*). At a concentration of 0.06% (*v*/*v*) caraway EO, microbial growth was observed, regardless of whether the brine and cheese were stored at 4 °C or 37 °C. At 4 °C storage, as expected, the microbial count was lower (brine samples—2.81 log_10_ CFU/mL and cheese samples—2.16 log_10_ CFU/g). It is well known that the storage of food products at low temperatures has an inhibitory effect on the growth of mesophilic bacteria. Upon the storage of the samples at 37 °C, a temperature that provides optimal conditions for microbial growth, the microbial count of *E. coli* reached mean values of 3.29 log_10_ CFU/mL in the brine and 2.20 log_10_ CFU/g in the cheese sample.

No viable cells were detected in the negative controls at both storage temperatures. In the positive controls stored at 37 °C and 4 °C, the microbial counts of β-D-glucuronidase-positive *E. coli* were 3.55 log_10_ CFU/mL in the brine; 2.51 log_10_ CFU/g in the cheese and 3.30 log_10_ CFU/mL in the brine; 2.46 log_10_ CFU/g in the cheese samples, respectively. At this control point, a concentration of 0.12% (*v*/*v*) caraway EO was determined to be the lowest effective concentration, achieving the complete inhibition of *E. coli* growth (<1 CFU/g in cheese and <10 CFU/mL in brine).

In evaluating the antimicrobial activity of caraway EO against *S. aureus* ATCC 29213 at the 3 h mark after sample preparation, the protocols outlined in ISO 6888-1:2022 were followed. At this control point, the results showed the absence of the preservative properties of the caraway essential oil in all samples, stored at 4 °C or 37 °C. For the set of samples stored at 4 °C, the *S. aureus* count in the brines was measured with average values between 3.68 and 4.02 log_10_ CFU/mL and in the cheese samples—2.40 and 2.95 log_10_ CFU/g. At 37 °C storage, microbial growth in the brine samples ranged from 4.16 log_10_ CFU/mL at a concentration of 5% (*v*/*v*) caraway EO to 4.45 log_10_ CFU/mL at the lowest tested concentration of 0.06% (*v*/*v*) (Table 2). In the cheese samples, *S. aureus* counts ranged from 3.27 log_10_ CFU/g at 5% (*v*/*v*) caraway EO to 3.85 log_10_ CFU/g at 0.06% (*v*/*v*), the lowest tested concentration.

In the positive controls, under both storage conditions, the microbial count in the brine samples was higher than in all the caraway EO samples. The same trend was observed in the cheese samples. No coagulase-positive staphylococci were detected in the negative control samples (<100 CFU/mL in brine; <10 CFU/g in cheese).

### 3.4. Microbiological Analysis at the 24th Hour After Sample Preparation

The second observation point was set at 24 h post-inoculation. Microbiological analysis for β-glucuronidase-positive *E. coli* yielded results consistent with those observed at the 3 h mark. Regardless of the storage temperature, all samples containing caraway EO concentrations ranging from 0.12% (*v/v*) to 5% (*v*/*v*) showed no viable cells. In samples stored at 4 °C, microbial growth was detected in both the brine (2.76 log_10_ CFU/mL) and cheese (2.20 log_10_ CFU/g) with 0.06% (*v*/*v*) caraway EO, as well as in the positive control samples. At 37 °C, in samples with 0.06% (*v*/*v*) caraway EO, viable cell counts were measured at 1.80 log_10_ CFU/mL in the brine, 1.37 log_10_ CFU/g in the cheese samples, and a significantly higher microbial count in the positive control sample. No microbial growth was detected in the negative control. The 24 h microbiological analysis confirmed that 0.12% (*v*/*v*) caraway EO was the lowest bactericidal concentration against *E. coli* (<1 CFU/g in cheese, <10 CFU/mL in brine) in both the tested brine and white brined cheese.

At the 24 h control point, the analysis for coagulase-positive staphylococci yielded results differing from those observed at the 3 h mark. At concentrations ranging from 0.25% (*v*/*v*) to 5% (*v*/*v*), caraway EO completely inhibited microbial growth in both brine and cheese samples (<10 CFU/g in cheese, <100 CFU/mL in brine) stored at 37 °C. In the samples cultured at 4 °C, the antimicrobial effect of the EO was even stronger as it demonstrated the inhibition of staphylococci in the cheese samples, even at a concentration of 0.12% (*v*/*v*). For these set of samples, microbial count (2.88 log_10_ CFU/g) was recorded in the cheese samples with a concentration 0.06% caraway EO, as well as in the brine samples with concentrations ranging from 0.06% to 0.12%, with values of 3.38 and 2.77 log_10_ CFU/mL, respectively. In the samples stored at 37 °C, microbial growth was observed in those with caraway EO concentrations ranging from 0.06% to 0.12% (*v*/*v*), with the average microbial count in the brines ranging from 2.69 to 2.79 log_10_ CFU/mL and in the cheese samples 2.58 to 2.73 log_10_ CFU/g.

In the positive controls, the microbial count was significantly higher than in all samples with the EO and presence of staphylococci, while no viable cells were detected in the negative controls. A concentration of 0.25% (*v*/*v*) caraway essential oil was identified as the minimum concentration required for complete inhibition of microbial growth 24 h after application in brine and white brined cheese.

### 3.5. Microbiological Analysis at the 168th Hour (7th Day) After Sample Preparation

The third control point for all samples was one week after their preparation. In the samples contaminated with *E. coli* and stored at 4 °C, the results showed stability in the preservative effect of caraway EO, with even an enhancement of its antimicrobial effects in the cheese samples—from a 0.12% (*v*/*v*) minimum bactericidal concentration in the previous measurements to 0.06% (*v*/*v*) on the 7th day, as indicated by the same parameter. In contrast, the brine samples showed no change, the lowest concentration at which no viable cells were detected was 0.12% (*v*/*v*). At a concentration of 0.06% (*v*/*v*), microbial growth was observed in the brine samples, with a mean value of 2.76 log_10_ CFU/mL. The set of samples stored at 37 °C showed a retention of the results from the previous two measurements—the minimum concentration of caraway EO at which the growth of *E. coli* was completely inhibited remained 0.12% (*v*/*v*). At a concentration of 0.06% (*v*/*v*), the presence of *E. coli* was detected, although at significantly lower levels compared to the positive controls. The presence of *E. coli* was not detected in the negative controls.

The conducted studies showed those seven days after sample preparation, the inhibitory effect of caraway EO against *S. aureus* remained unchanged compared to the 24 h control point. Samples stored at 4 °C confirmed 0.12% (*v*/*v*) as the lowest bactericidal concentration of the EO in the cheese samples and 0.25% (*v*/*v*) in the brine samples. For the samples stored at 37 °C, this effect was achieved at a concentration of 0.25% caraway essential oil in both cases. For the samples with the EO, where the presence of staphylococci was detected, the microbial count varied from 2.36 to 2.70 log_10_ CFU per mL or gram sample, depending on the type of sample and storage temperature.

Similar to all the presented samples, the microbial counts in the positive controls were significantly higher than those in the samples containing essential oil and staphylococci. All results from the negative controls were negative for the presence of coagulase-positive staphylococci.

**Figure 2:**
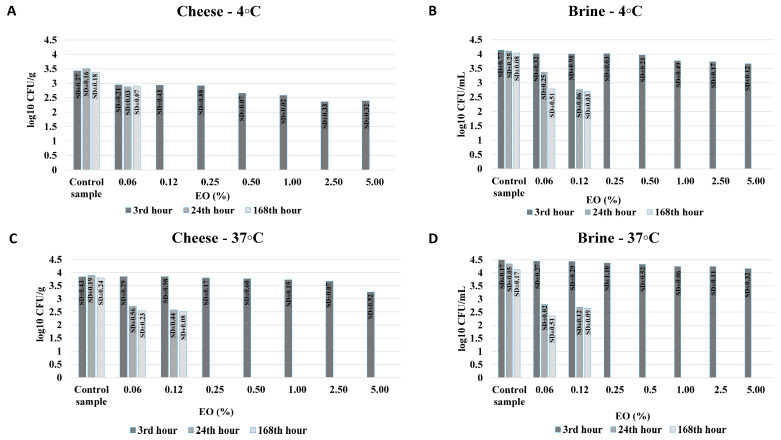
Preservative potential of caraway essential oil (EO) in brine and cheese stored against *Staphylococcus aureus* ATCC 29213. (**A**,**B**)—samples, stored at 4 °C; (**C**,**D**)—samples, stored at 37 °C. Note: All log_10_ CFU/g and log_10_ CFU/mL values are presented as mean values from three consecutive experiments.

**Figure 3:**
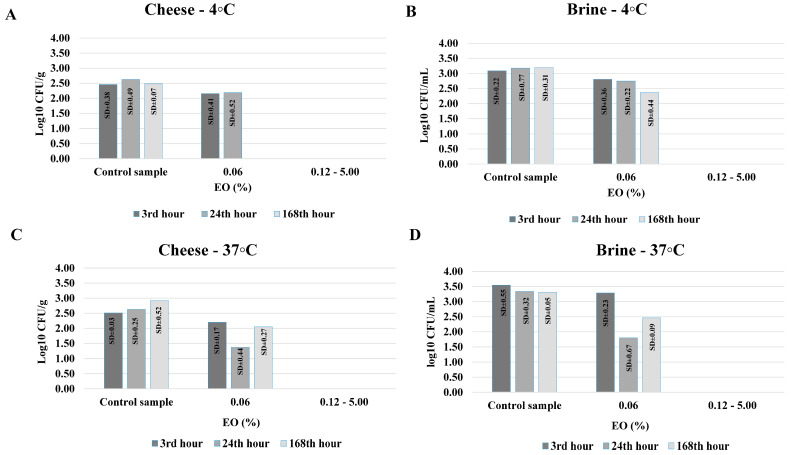
Preservative potential of caraway essential oil (EO) in cheese and brine against *Escherichia coli* ATCC 25922. (**A**,**B**)—samples, stored at 4 °C; (**C**,**D**)—samples, stored at 37 °C. Note: All log_10_ CFU/g and log_10_ CFU/mL values are presented as mean values from three consecutive experiments.

The complete results of the antimicrobial activity of CEO in brine are presented in Figure 2 and Figure 3.

### 3.6. Biochemical Identification of Escherichia coli-Positive Samples

**Figure 4:**
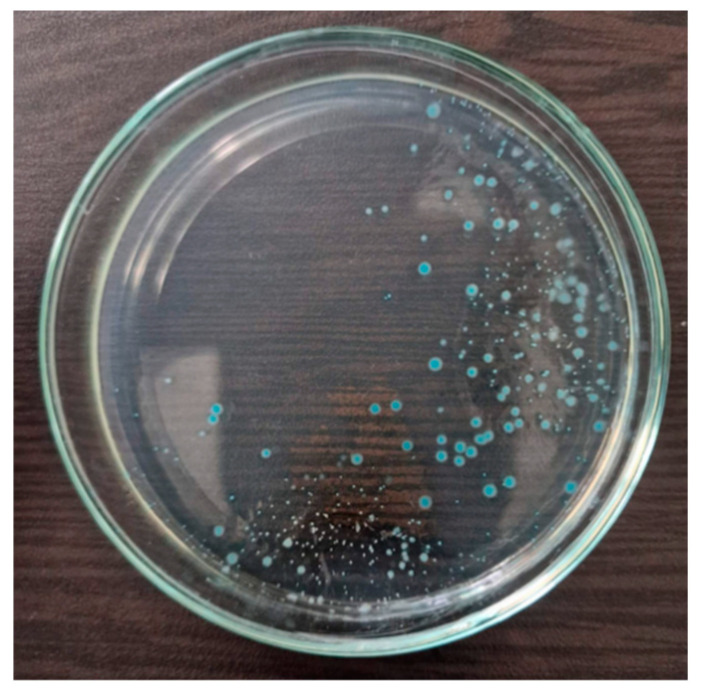
Cultivation of *Escherichia coli* ATCC 25922 on TBH agar. Typical bacterial colonies of β-D-glucuronidase-positive *Escherichia coli* on TBH agar are presented in blue to blue-green color.

From all the samples presented in this work, in which typical for *E. coli* growth was detected (Figure 4), at least three colonies from each Petri dish were subjected to biochemical identification. All samples exhibited the characteristic biochemical profile of *E. coli*—positive for lactose and glucose fermentation, negative for hydrogen sulfide production, and positive for gas production. The indole test was also positive for all tested colonies.

### 3.7. Detection of Coagulase Enzymes in Samples Positive for Staphylococcus aureus

**Figure 5:**
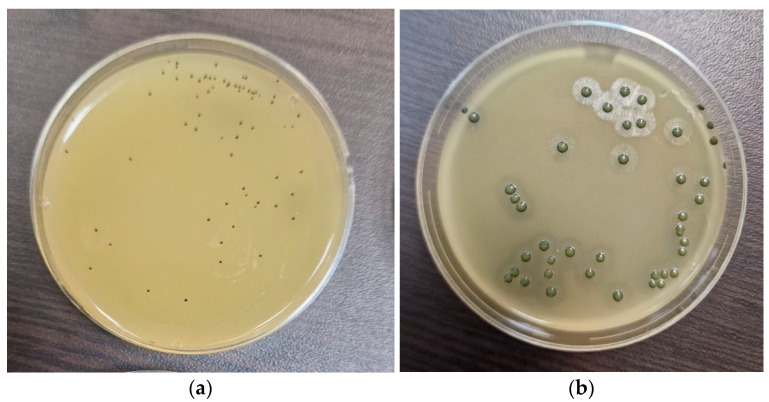
The results obtained from a typical growth of *Staphylococcus aureus* ATCC 29213 in Baird Parker agar: (**a**) colonies after 24 h of cultivation without the opalescent ring formed for the typical colonies of coagulase-positive staphylococci around the black colonies; (**b**) colonies after 48 h of cultivation, with a clearly pronounced opalescent zone around the bacterial colonies.

A coagulase test was performed for all samples described above, where the growth of staphylococci was detected. From all Baird Parker plates with typical black colonies, with or without an opalescent zone surrounding them (Figure 5), at least three colonies per sample were subjected to testing for the presence of plasma-coagulase enzymes. All tested colonies were positive for coagulase-positive staphylococci as they induced the coagulation of rabbit plasma.

### 3.8. Assessment of the Influence of Caraway Oil on the Organoleptic Characteristics of Cheese

One of the objectives of this study was to investigate the impact of brine containing 0.25% (*v*/*v*) caraway EO on the organoleptic characteristics of white brined cheese during storage. The baseline assessment of these parameters, prior to the addition of essential oil, is presented in Table 1, showing a total score of 99.63.

After 168 h (7 days) of storage at 4 °C, the evaluation indicated that the addition of caraway EO had no impact on any of the criteria used for scoring—taste and aroma, texture, the appearance of the pieces, cut surface, structure and color, and brine condition. Following the addition of caraway EO and the subsequent seven-day storage period, the organoleptic characteristics of the cheese were reassessed, without significant change in the total score—from 99.63 to 99.51. The obtained results are presented in Table 3.

### 3.9. Correlation Analysis of Antimicrobial Activity

A correlation analysis was conducted to examine the relationship between the concentration of caraway essential oil (EO) and microbial growth in cheese and brine samples. The analysis focused on data from the third hour of incubation as it reflects the early effect of the oil before complete microbial inhibition. At later time points (24 and 168 h), many samples exhibited total inhibition, potentially distorting the results (Table 4).

For the samples stored at 37 °C, the correlation analysis revealed a strong negative correlation between the concentration of caraway EO and the microbial count of *S. aureus*. This effect was statistically significant in both cheese (r = −0.976, *p* = 0.00017) and brine (r = −0.938, *p* = 0.0018), demonstrating a dose-dependent inhibitory effect of the EO. In contrast, the relationship between EO concentration and the microbial count of *E. coli* showed negative correlations in cheese (r = −0.471, *p* = 0.286) and brine (r = −0.472, *p* = 0.285), but these were not statistically significant. The weaker correlation for *E. coli* suggests an early bactericidal effect at lower concentrations as most values were recorded as zero.

When samples were stored at 4 °C, a similar pattern was observed. There was a strong negative correlation between caraway EO concentration and *S. aureus* growth (cheese: r = −0.823, *p* = 0.023; brine: r = −0.877, *p* = 0.010), indicating significant bacterial reduction. However, due to the limited data points for *E. coli*, statistical conclusions were uncertain (cheese: r = −1.0, *p* = 1.0; brine: r = −1.0, *p* = 1.0) despite observed growth inhibition at increasing EO concentrations.

In conclusion, caraway EO exhibited a strong dose-dependent inhibitory effect on *S. aureus* at both 37 °C and 4 °C, while its impact on *E. coli* was evident but difficult to assess due to early bactericidal effects. The results demonstrate similar antimicrobial trends in both cheese and brine samples.

### 3.10. An Escherichia coli Model Network

The largest cluster (red in the center of the network) was formed around DNA topological change, centered on the *DNA gyrase subunits A and B* (*gyrA*, *gyrB*). These subunits belong to *type II topoisomerases*, which negatively supercoil double-stranded DNA in an ATP-dependent manner to maintain chromosomes in an underwound state. The network analysis has shown that these two *DNA gyrase* subunits are targets of EO. Additionally, the *DNA gyrase subunits*, as well as *DNA topoisomerase 4 subunit A* (*parC*) and *DNA topoisomerase 4 subunit B* (*parE*), have also been identified as EO targets.

The second largest cluster is formed around Quorum Sensing (QS, yellow, located in the bottom right of the network and separated below the center by a dotted line labeled “Quorum Sensing”). This cluster includes *sdiA*, *qseC*, *qseB*, and *LuxS*.

**Figure 6:**
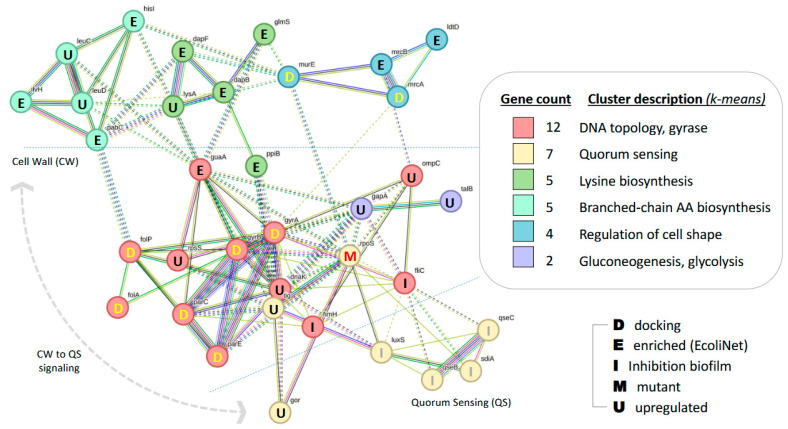
A network targeted by essential oil (carvone/limonene) infers multiple circuits for signaling.

The remaining nodes (genes and proteins) are associated with various functions at the bacterial cell wall, such as lysine biosynthesis, branched-chain amino acid biosynthesis, and cell shape regulation. These nodes are colored in shades of green, positioned at the top of the network, and separated above the center by a dotted line labeled “Cell Wall” (CW). Two straight lines have been added to further delineate the clustering results. A bidirectional side arrow indicates the general direction of signaling received from the external environment, which affects the circuits of the cell wall (CW) and passes through DNA-driven circuits (*Gyr* and *Rpo*) before reaching the Quorum Sensing (QS) circuit. Capital letters centered within the nodes indicate their source, which is listed alphabetically in the bottom right corner. A bracket between “D” (for docking source) and “U” (for upregulation source) connects twenty-one nodes gathered in the first step described in the Methods section (Figure 6).

## 4. Discussion

The present study investigates the antimicrobial activity of caraway EO in brine with reduced salt content, demonstrating its potential for controlling pathogenic microflora in the brine of white brined cheese (Figure 1). The antimicrobial properties of caraway EO and its main components, carvone and limonene, have been confirmed in numerous recent scientific studies [43,44,45,46]. In our study, we focused on using caraway EO as an active component for post-preservation, which could also allow for a certain reduction in sodium chloride content in the solution.

The preservative potential of caraway EO against the proliferation of *S. aureus* ATCC 29213 and *E. coli* ATCC 25922 in cheese and brine, stored at 4 °C and 37 °C, is presented in Figure 2 and Figure 3.

The two sets of samples stored at 37 °C allowed us to assess both the antimicrobial effectiveness of caraway EO at a higher storage temperature and its action during optimal microbial growth. The results obtained from these samples showed that, in the positive control stored at 37 °C, the bacterial growth of *S*. *aureus* remained stable throughout the observation period and even increased over time. These results indicate that a 2% sodium chloride concentration in the brine allows the growth of *S*. *aureus*, which is an expected outcome, as its tolerance to high salt concentrations has been documented [47]. Previous studies have shown that *Staphylococcus spp*. can survive at salt concentrations up to 15% [47,48].

In the samples containing caraway EO, the CFU values of *S*. *aureus* were consistently lower than those in the positive control, although the complete inhibition of microbial growth was not observed at any concentration of the essential oil during the first control point (the third hour). However, the applied correlation analysis demonstrated that as the concentration of the oil increased, the number of *S*. *aureus* significantly decreased, and this effect was statistically significant (cheese: r = −0.953, *p* = 0.0009; brine: r = −0.938, *p* = 0.0018).

At the 24-hour and 7-day measurements, all samples with caraway EO concentrations of 0.25% (*v*/*v*) or higher were proven effective in inhibiting the growth of *S*. *aureus*, and the antimicrobial effects remained stable throughout the entire observation period. The slower onset of caraway EO activity against *S*. *aureus* can likely be explained by the findings of Nogueira et al., 2021 [48]. They report that carvone, the main component of caraway EO, initially has an effect contrary to expectations in physiological solutions, increasing the survival of *S*. *aureus* compared to bacterial controls. These compounds may positively influence the content of osmotically active dissolved substances or the permeability of the bacterial membrane, leading to an increased survival rate of microorganisms in hypertonic environments [48].

In the same set of samples stored at 37 °C, monitoring the effect of caraway EO on the development of *E. coli* ATCC 25922 also revealed a decrease in bacterial count as the oil concentration increased. Negative correlations were observed in all cheese and brine samples, although statistical significance was not reached (r = −1.0, *p* = 1.0), likely due to the rapid inhibition of microbial growth after the first tested concentration. In the sample containing 0.06% (*v*/*v*) caraway EO, which was identified as the only ineffective concentration, a significant reduction in CFUs was recorded at all control points, especially in brine. At concentrations of 0.12% (*v*/*v*) and higher, the growth of *E*. *coli* was completely inhibited, and these results remained stable throughout the entire seven-day observation period.

By storing two analogous sets of samples at 4 °C, we were able to assess the effect of lower storage temperatures on the antimicrobial activity of caraway EO. The results showed that the 4 °C temperature itself slows bacterial growth, which was an expected outcome. Focusing on *S. aureus*, we observed that at 4 °C, the oil was more effective compared to storage at 37 °C—even at low concentrations, a stronger bactericidal effect was noted. Furthermore, the long-term preservative activity (7 days) remained stable.

The main observations for *E. coli* also demonstrated that at 4 °C, the EO was more effective, supporting the conclusion that low temperature enhances the antimicrobial effect of caraway EO, likely assisted by the slowed bacterial metabolism. These results support the use of caraway EO as a preservative for products stored at low temperatures, which is crucial for its application in the food industry.

Statistically significant results demonstrated strong antimicrobial effects against *S. aureus*, while an even more rapid and effective action was observed against *E. coli*, an indicator of hygienic contamination in the food industry.

Despite this, the limited number of data points prevented us from establishing a statistically significant strong negative correlation between the essential oil concentration and the microbial count of *E. coli*. To further investigate the potential antimicrobial mechanism of action, a literature review was conducted on molecular docking simulations examining the effects of carvone and limonene, the major bioactive components of caraway essential oil, on key structural bacterial proteins of *E. coli*.

The modeled network and its inferred clusters reveal a functional sub-segmentation akin to the organization of a bacterial cell. The antibacterial action of EOs is primarily attributed to phenolic compounds, which disrupt cell membrane integrity and biofilm formation. Additionally, EOs can interfere with bacterial communication, impairing their ability to coordinate with their environment and maintain survival. The network model suggests that signals from the outer membrane trigger intracellular pathways, ultimately inhibiting Quorum Sensing (QS).

Key nodes in the network are linked to cell wall formation and cell shape regulation, such as the L-lysine biosynthesis pathway and enzymes like Diaminopimelate epimerase (dapF). A crucial node is GMP synthetase (guaA), involved in DNA replication and energy processes, and it connects with proteins like DNA gyrase and topoisomerases, all targets of EOs. 

The network also includes RpoS, a master regulator of stress response, and DnaK, a heat-shock protein linked to DNA replication and biofilm formation. Crl, a biofilm regulator, interacts with RpoS to facilitate stress resistance. SdiA, a LuxS family transcription factor, mediates QS in *E. coli*, which EOs can inhibit, offering a promising strategy to prevent biofilm formation. In pathogenic *E. coli*, QS controls virulence factor expression and motility, crucial for biofilm formation, chronicity, and antibiotic resistance. EOs, such as limonene, offer potential in combating antimicrobial resistance by targeting multiple cellular processes, from membranes to QS suppression.

The antimicrobial properties of caraway EO are determined by its chemical composition. In the EO, used in our study, 13 compounds were identified, among which two compounds remained unidentified (Table 1). Some authors suggest that there are more components in caraway EO [49,50,51,52,53]. It was established that the EO is constituted mainly of monoterpenes. In our experiment, the main components were carvone and limonene, whose mixture constituted almost 100% of total oil composition. It can be concluded that the studied sample meets the Eur.Ph [54] standard for the content of these components, which should be between 50 and 65% for carvone and 30 and 45% for limonene, respectively.

Scientific data on the antimicrobial activity of caraway EO against *S. aureus* are available in a study by Liu C et al. They investigate the impact of the EO extracted from *Carum carvi* L. seeds on *S. aureus* and the related mechanism of action. Their results show that the essential oil significantly inhibits biofilm formation in staphylococci cells. Their amino acid metabolism was found to be significantly affected by the essential oil, with 63 metabolites being impacted. In conclusion, the authors report that caraway seed essential oil has potent antimicrobial properties and demonstrates promising potential for use in food and pharmaceuticals [32].

Recent studies by Abd El-Aziz et al. and Rys et al. explore the potential of caraway oil and caraway-oil-loaded bio-nano emulsions in dairy products, specifically cheese, and find that caraway EO effectively inhibited the growth of spoilage microorganisms, thereby enhancing the cheese’s shelf life and safety [27,28].

Equally important is how the essential oil affects the lactic acid bacteria in dairy products. The natural cheese microbiota and starter cultures are responsible for fermentation and for imparting the desired sensory characteristics to the product, so maintaining their species composition, microbial count, and suitable low pH for their growth is crucial. Solid scientific data indicate that several EOs with antimicrobial activity against pathogens do not affect the population of lactic acid bacteria in the same concentration [55,56], and the product’s pH does not significantly change [57]. A research team from Bulgaria, Trifonov et al., studied the impact of fruits and caraway EO on the normal development of the lactobacillus population in white brined cheese, as well as the effect of the EO on the organoleptic characteristics of the product. According to their results, the additives had a beneficial effect on acid formation, the development of lactic acid bacteria, and the key organoleptic attributes [58].

## 5. Conclusions

The findings of our study demonstrated that caraway EO exhibits a potent inhibitory effect in brine and white brined cheese. The essential oil shows potential as an effective preservative for cheese, with the ability to completely suppress microbial growth during hygienic contamination with *E. coli*, even in cases of high microbial loads. Analyzing the results from all the tested samples, we found that 0.12% caraway EO is a minimal bactericidal concentration against *Escherichia coli*, while for *S. aureus*, similar effects were achieved at 0.25% (*v*/*v*) in both brine and cheese.

Regarding *S. aureus*, caraway EO displayed antimicrobial activity with a delayed effect. In the analysis of samples conducted at the 3 h mark, the EO did not completely inhibit microbial growth. However, by the 24 h mark, microbial counts in the samples with 0.25% (*v*/*v*) caraway EO and higher concentrations were effectively suppressed. Additionally, one of the most significant findings was the retained antimicrobial effectiveness up to the 7th day of storage, which is promising for the use of caraway EO as a preservative.

## Figures and Tables

**Table 1:** Microbial safety, organoleptic, and physicochemical characteristics of the cheese samples used in the study.

Microbial Safety		
Presence of glucuronidase-positive *E. coli*	Negative	
Presence of coagulase-positive staphylococci	Negative	
Presence of *Salmonella* spp.	Negative	
Presence of *Listeria monocytogenes*	Negative	
**Organoleptic characteristics—100-point scale**		
Taste and smell	44.75 ± 0.43	Total score 99.63
Consistency	19.88 ± 0.33	
Appearance of pieces	5.00 ± 0.00	
Cut surface, structure, and color	15.00 ± 0.00	
Condition of brine	5.00 ± 0.00	
Packaging and labeling	10.00 ± 0.00	
**Physicochemical characteristics**		
Total titratable acidity (°T)	232.6 ± 2.5	
Content of table salt (%)	3.97 ± 0.6	
Content of dry matter (%)	96.3 ± 2.6	
Fat content (%)	51.7 ± 0.7	
Degree of ripeness (%)	70.0 ± 0.0	

**Table 2:** Composition of the essential oil of *Carum carvi*.

Name	%TIC	RT
ß-Myrcene	0.09	11.16
Limonene	28.19	12.52
(E)-p-Mentha-2,8-dien-1-ol	0.13	15.36
(Z)-p-Mentha-2,8-dien-1-ol	0.10	15.81
(Z)-p-Mentha-8-en-2-one	0.41	17.63
(E)-p-Mentha-8-en-2-one	0.23	17.80
(E)-Carveol	0.22	18.49
(Z)-Carveol	0.25	18.86
Carvone	69.80	19.22
p-Mentha-1,8-dien-7-al	0.23	19.91
Limonene aldehyde	0.14	21.81
ß-Caryophyllene	0.08	23.68
Caryophyllene oxide	0.09	27.67

Legend: %TIC—percentage of total ion current. RT—retention time.

**Table 3:** Organoleptic characteristics of cheese and brine enriched with caraway EO 100-point scale.

Taste and smell	45.00 ± 0.00	Total score 99.51
Consistency	19.75 ± 0.43	
Appearance of pieces	5.00 ± 0.00	
Cut surface, structure, and color	14.88 ± 0.33	
Condition of brine	5.00 ± 0.00	
Packaging and labeling	9.88 ± 0.33	

**Table 4:** Statistical correlation analysis of caraway EO on microbial growth.

Storage Temperature	Bacterial Strain	Sample Type	Correlation Coefficient (r)	*p*-Value	Statistical Significance
37 °C	*S. aureus*	Cheese	−0.976	0.00017	Yes
37 °C	*S. aureus*	Brine	−0.938	0.0018	Yes
37 °C	*E. coli*	Cheese	−0.471	0.286	No
37 °C	*E. coli*	Brine	−0.472	0.285	No
4 °C	*S. aureus*	Cheese	−0.823	0.023	Yes
4 °C	*S. aureus*	Brine	−0.877	0.010	Yes
4 °C	*E. coli*	Cheese	−1.000	1.000	No (Limited Data)
4 °C	*E. coli*	Brine	−1.000	1.000	No (Limited Data)

## Data Availability

The original contributions presented in the study are included in the article/Supplementary Materials, further inquiries can be directed to the corresponding author.

## Supplementary Materials

The following supporting information can be downloaded at: https://www.mdpi.com/article/10.3390/foods14081297/s1, Table S1: Study of the preservative potential of brine (2% sodium chloride) enriched with caraway essential oil against *Staphylococcus aureus* ATCC 29213 и *Escherichia coli* ATCC 25922, in samples stored at 37 °C; Table S2: Study of the preservative potential of brine (2% sodium chloride) enriched with caraway essential oil against *Staphylococcus aureus* ATCC 29213 и *Escherichia coli* ATCC 25922, in samples stored at 4 °C
