# Supplementary material for: Caraway Essential Oil as a Post-Preservative Agent in Low-Salt Cheese Brine
Source: Foods. 2025 Apr 8;14(8):1297. doi: 10.3390/foods14081297 (PMC12026341; doi:10.3390/foods14081297)
Supplement: Supplementary file 1 [file foods-14-01297-s001.zip › foods-3557983-supplementary.pdf]

Table S1. Study of the preservative potential of brine (2% sodium chloride) enriched with caraway essential oil against *Staphylococcus aureus* ATCC 29213 и *Escherichia coli* ATCC 25922, in samples stored at 37∘C.

Control points		Control samples		Caraway essential oil added to cheese brine (% (v/v))						
		(-) control	(+) control	0.06	0.12	0.25	0.5	1	2.5	5
Staphylococcus aureus ATCC 29213 (log_{10} CFU/g / log_{10} CFU/mL)										
3th hour	**C**	NG	3.84 ±0.43	3.85±0.29	3.90±0.98	3.80±0.17	3.77±0.60	3.73±0.15	3.67±0.07	3.27±0.52
	**B**	NG	4.52±0.17	4.45±0.27	4.40±0.29	4.38±1.10	4.34±0.52	4.25±0.06	4.26±0.11	4.16±0.32
24th hour	**C**	NG	3.91±0.19	2.73±0.56	2.58±0.44	NG	NG	NG	NG	NG
	**B**	NG	4.35±0.05	2.79±0.02	2.69±0.12	NG	NG	NG	NG	NG
168th hour (7th day)	**C**	NG	3.81±0.24	2.55±0.23	2.51±0.08	NG	NG	NG	NG	NG
	**B**	NG	4.13±0.17	2.36±0.51	2.65±0.09	NG	NG	NG	NG	NG
Escherichia coli ATCC 25922 (log_{10} CFU/g / log_{10} CFU/mL)										
3th hour	**C**	NG	2.51±0.03	2.20±0.17	NG	NG	NG	NG	NG	NG
	**B**	NG	3.55±0.55	3.29±0.23	NG	NG	NG	NG	NG	NG
24th hour	**C**	NG	2.63 ±0.25	1.37±0.44	NG	NG	NG	NG	NG	NG
	**B**	NG	3.34 ±0.32	1.80±0.67	NG	NG	NG	NG	NG	NG
168th hour (7th day)	**C**	NG	2.92 ±0.52	2.06±0.27	NG	NG	NG	NG	NG	NG
	**B**	NG	3.32 ±0.05	2.47±0.09	NG	NG	NG	NG	NG	NG

CFU – colony forming units; NG – negative for growth; C – cheese sample; B – brine sample

(-) control contains 2% sodium chloride brine and cheese; (+) control contains 2% sodium chloride brine, cheese and 0.5MF microbial strain.

Table S2. Study of the preservative potential of brine (2% sodium chloride) enriched with caraway essential oil against *Staphylococcus aureus* ATCC 29213 и *Escherichia coli* ATCC 25922, in samples stored at 4∘C.										
Control points		Control samples		Caraway essential oil added to cheese brine (% (v/v))						
		(-) control	(+) control	0.06	0.12	0.25	0.50	1.00	2.50	5,00
Staphylococcus aureus ATCC 29213 (log10 CFU/g / log10 CFU/mL)										
3th hour	**C**	NG	3.43±0.27	2.95±0.21	2.94±0.43	2.92±0.88	2.66±0.07	2.59±0.02	2.37±0.33	2.40±0.32
	**B**	NG	4.14±0.77	4.02±0.32	4.01±0.91	4.02±0.03	3.97±0.21	3.78±0.49	3.74±0.17	3.68±0.12
24th hour	**C**	NG	3.51±0.16	2.88±0.03	NG	NG	NG	NG	NG	NG
	**B**	NG	4.11±0.25	3.38±0.25	2.77±0.06	NG	NG	NG	NG	NG
168th hour (7th day)	**C**	NG	3.39±0.18	2.91±0.07	NG	NG	NG	NG	NG	NG
	**B**	NG	4.04±0.08	2.79±0.51	2.70±0.03	NG	NG	NG	NG	NG
Escherichia coli ATCC 25922 (log10 CFU/g / log10 CFU/mL)										
3th hour	**C**	NG	2.46±0.38	2.16±0.41	NG	NG	NG	NG	NG	NG
	**B**	NG	3.09±0.22	2.80±0.36	NG	NG	NG	NG	NG	NG
24th hour	**C**	NG	2.63±0.49	2.20±0.52	NG	NG	NG	NG	NG	NG
	**B**	NG	3.18±0.77	2.76±0.22	NG	NG	NG	NG	NG	NG
168th hour (7th day)	**C**	NG	2.48±0.07	NG	NG	NG	NG	NG	NG	NG
	**B**	NG	3.20±0.31	2.37±0.44	NG	NG	NG	NG	NG	NG

CFU – colony forming units; NG – negative for growth; C – cheese sample; B – brine sample

(-) control contains 2% sodium chloride brine and cheese; (+) control contains 2% sodium chloride brine, cheese and 0.5MF microbial strain.

The complete results of the antimicrobial activity of brine (2% sodium chloride) enriched with caraway essential oil against *Staphylococcus aureus* ATCC 29213 and *Escherichia coli* ATCC 25922 are presented in Tables S1 and S2.
